# The genome sequence of the Large Brook Dun, *Ecdyonurus torrentis *(Kimmins, 1942)

**DOI:** 10.12688/wellcomeopenres.20149.1

**Published:** 2023-10-18

**Authors:** Andrew Farr, Craig Macadam, Natural History Museum Genome Acquisition Lab, Darwin Tree of Life Barcoding collective, Wellcome Sanger Institute Tree of Life programme, Wellcome Sanger Institute Scientific Operations: DNA Pipelines collective, Tree of Life Core Informatics collective, Darwin Tree of Life Consortium

**Affiliations:** 1 Independent researcher, Hailsham, England, UK; 2 Buglife – The Invertebrate Conservation Trust, Stirling, Scotland, UK

**Keywords:** Ecdyonurus torrentis, Large Brook Dun, genome sequence, chromosomal, Ephemeroptera

## Abstract

We present a genome assembly from an individual female *Ecdyonurus torrentis* (the Large Brook Dun; Arthropoda; Insecta; Ephemeroptera; Heptageniidae). The genome sequence is 503.2 megabases in span. Most of the assembly is scaffolded into 11 chromosomal pseudomolecules, including the X sex chromosome. The mitochondrial genome has also been assembled and is 15.69 kilobases in length.

## Species taxonomy

Eukaryota; Metazoa; Eumetazoa; Bilateria; Protostomia; Ecdysozoa; Panarthropoda; Arthropoda; Mandibulata; Pancrustacea; Hexapoda; Insecta; Dicondylia; Pterygota; Palaeoptera; Ephemeroptera; Setisura; Heptageniidae; *Ecdyonurus*; *Ecdyonurus torrentis* (Kimmins, 1942) (NCBI:txid2014018).

## Background

**Figure 1.**
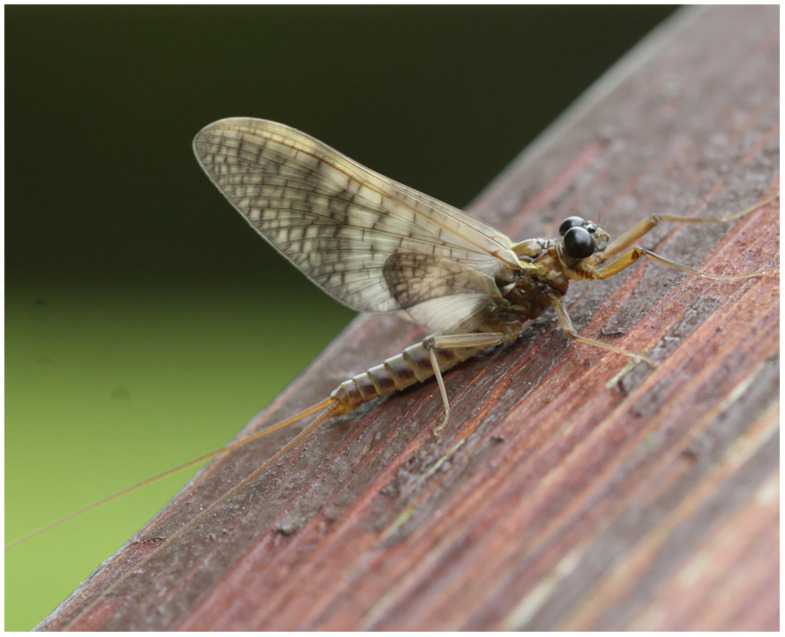
Photograph of *Ecdyonurus torrentis* (not the specimen used for genome sequencing) by David Nicholls, Lea Meadows, 22 May 2015.

*Ecdyonurus torrentis* ( Figure 1) is a western Palearctic species found across central Europe from France to Ukraine. It is absent from Fennoscandia. It is found throughout Britain and Ireland, generally in northern and western areas, although there are scattered records from the south-east of England and the south of Ireland ( GBIF Secretariat, 2023).

This species is considered a eurytherm and is typically found in the upper and middle reaches of watercourses ( Buffagni *et al.*, 2009). It is found at a range of altitudes, from upland streams to lowland watercourses. Larvae of *E. torrentis* are typically found in riffle areas of rivers and streams. They are usually found clinging to submerged stones, although they may swim if disturbed ( Elliott *et al.*, 1988).

*Ecdyonurus torrentis* is univoltine, overwintering as larvae and adults emerging between May and September ( Elliott *et al.*, 1988; Landa, 1968; Macan, 1957; Sowa, 1975; Sowa, 1979; Wise, 1980). In some years adults can be found as early as March. The flight period is often related to the altitude of the emergence site. In upstream reaches the flight period can last for up to three months, whereas at lower altitudes the flight period may be as short as one month ( Wise, 1980). The larvae feed by either scraping algae from the substrate or gathering fine particulate organic matter from the sediment ( Elliott *et al.*, 1988).

The genome sequence for *Ecdyonurus torrentis* will aid in understanding the biology, physiology and ecology of the species and the relationship between this species and other *Ecdyonurus* species in Europe. 

## Genome sequence report

The genome was sequenced from one female *Ecdyonurus torrentis* collected from River Rye, Yorkshire, UK (54.21, –0.98). A total of 67-fold coverage in Pacific Biosciences single-molecule HiFi long reads and 76-fold coverage in 10X Genomics read clouds were generated. Primary assembly contigs were scaffolded with chromosome conformation Hi-C data. Manual assembly curation corrected 200 missing joins or mis-joins and removed 45 haplotypic duplications, reducing the assembly length by 1.77% and the scaffold number by 42.45%, and increasing the scaffold N50 by 1.06%.

**Figure 2.**
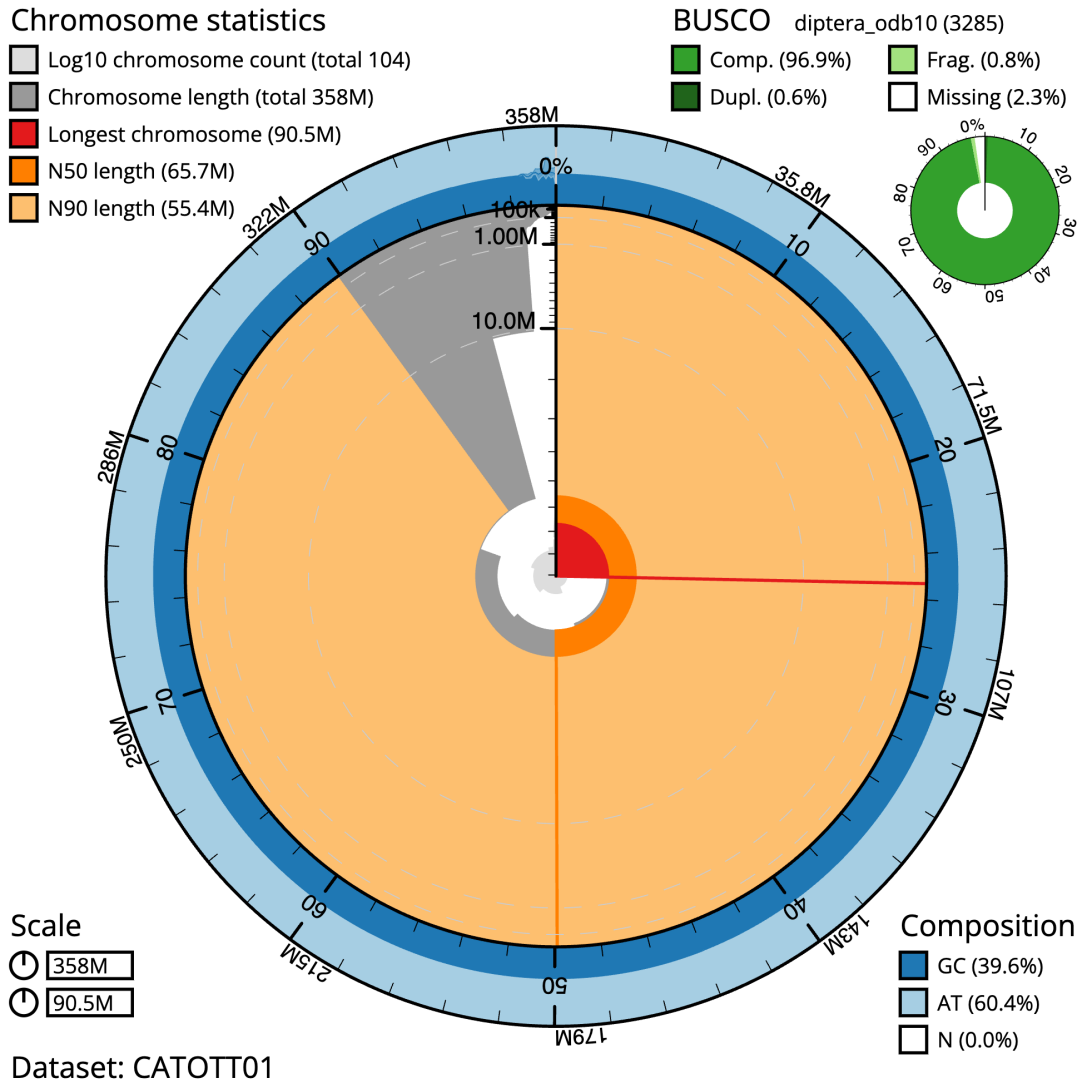
Genome assembly of *Ecdyonurus torrentis*, ieEcdTorr1.1: metrics. The BlobToolKit Snailplot shows N50 metrics and BUSCO gene completeness. The main plot is divided into 1,000 size-ordered bins around the circumference with each bin representing 0.1% of the 357,657,189 bp assembly. The distribution of scaffold lengths is shown in dark grey with the plot radius scaled to the longest scaffold present in the assembly (90,518,639 bp, shown in red). Orange and pale-orange arcs show the N50 and N90 scaffold lengths (65,719,635 and 55,360,379 bp), respectively. The pale grey spiral shows the cumulative scaffold count on a log scale with white scale lines showing successive orders of magnitude. The blue and pale-blue area around the outside of the plot shows the distribution of GC, AT and N percentages in the same bins as the inner plot. A summary of complete, fragmented, duplicated and missing BUSCO genes in the diptera_odb10 set is shown in the top right. An interactive version of this figure is available at https://blobtoolkit.genomehubs.org/view/idCriRanu1.1/dataset/CATOTT01/snail.

**Figure 3.**
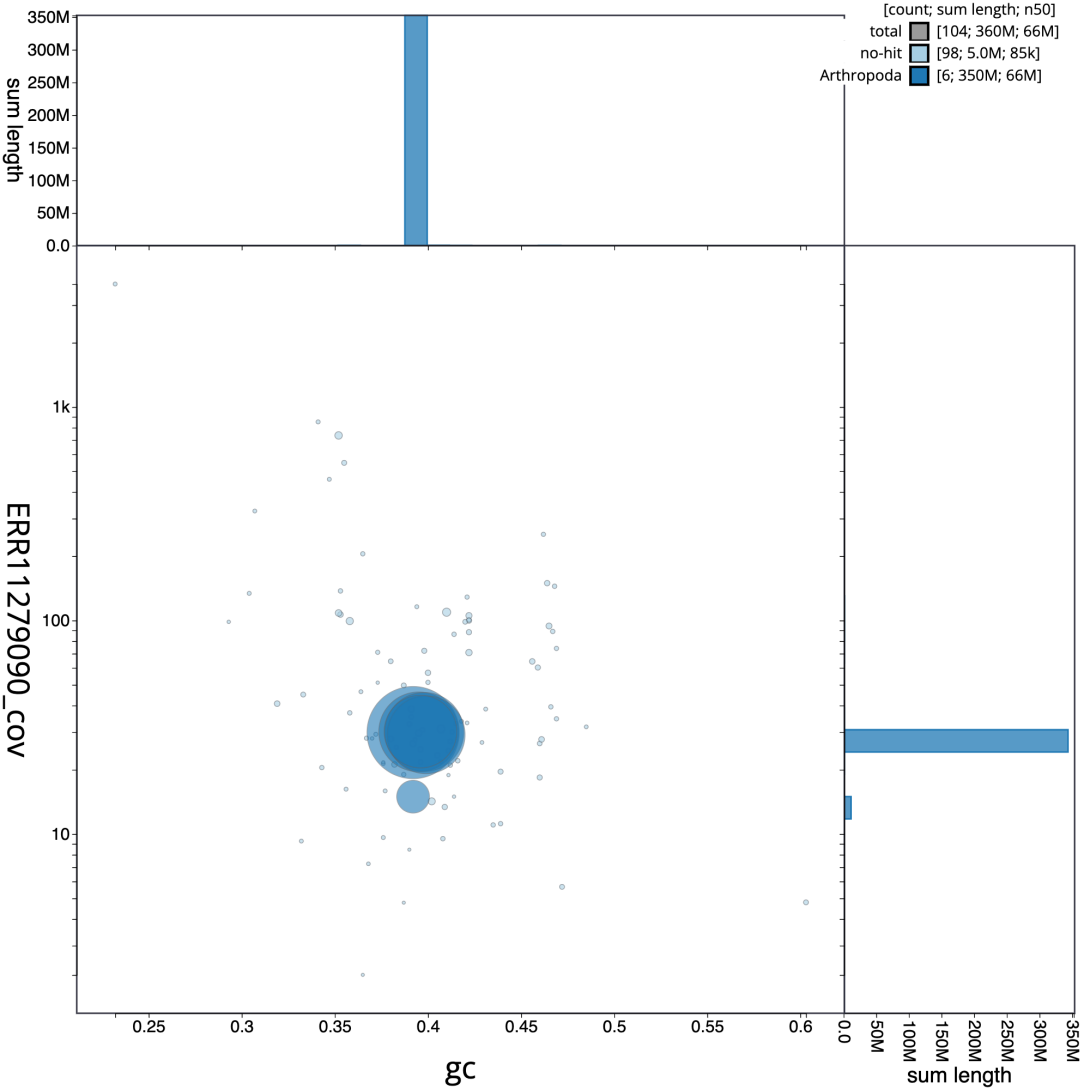
Genome assembly of *Ecdyonurus torrentis*, ieEcdTorr1.1: BlobToolKit GC-coverage plot. Scaffolds are coloured by phylum. Circles are sized in proportion to scaffold length. Histograms show the distribution of scaffold length sum along each axis. An interactive version of this figure is available at https://blobtoolkit.genomehubs.org/view/idCriRanu1.1/dataset/CATOTT01/blob.

**Figure 4.**
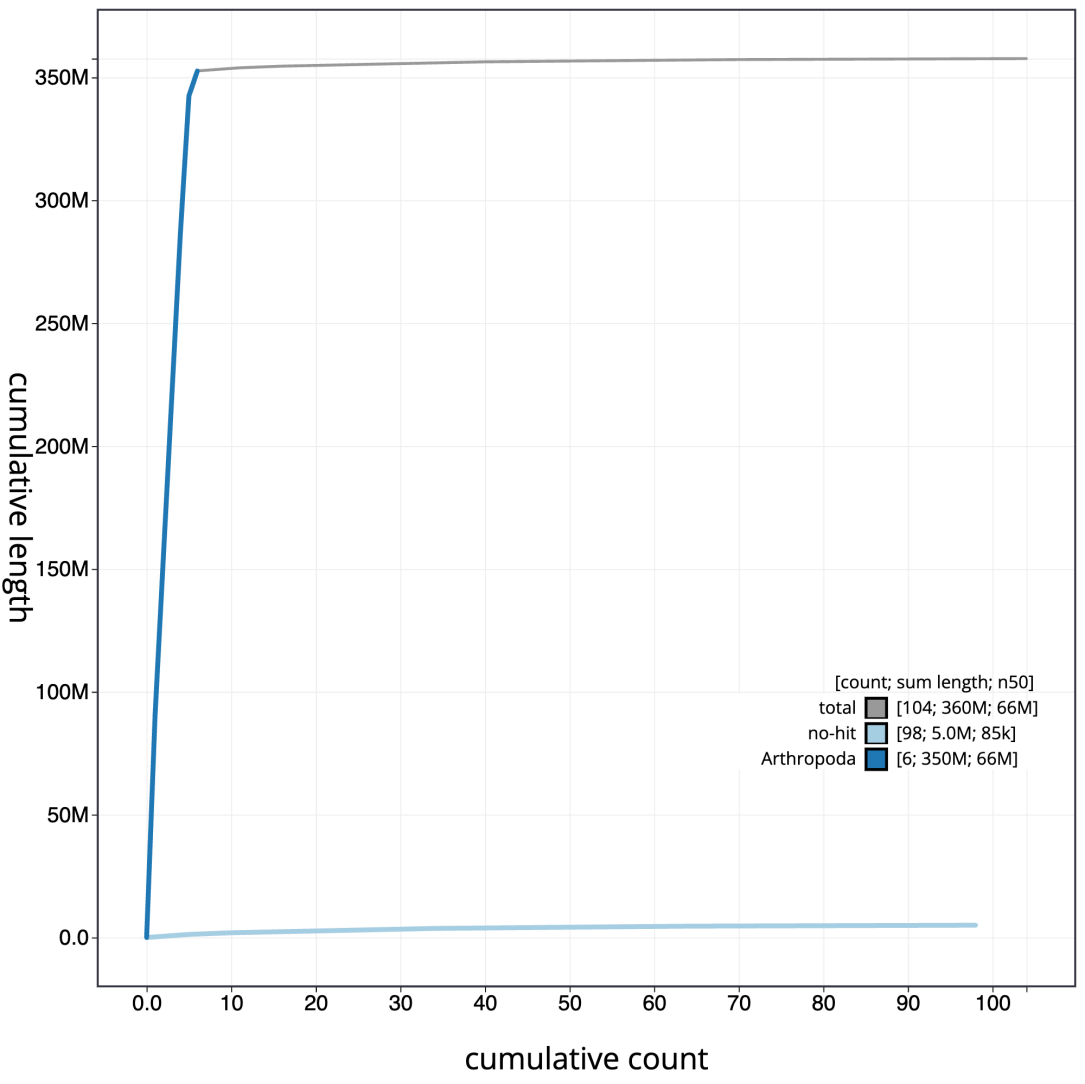
Genome assembly of *Ecdyonurus torrentis*, ieEcdTorr1.1: BlobToolKit cumulative sequence plot. The grey line shows cumulative length for all scaffolds. Coloured lines show cumulative lengths of scaffolds assigned to each phylum using the buscogenes taxrule. An interactive version of this figure is available at https://blobtoolkit.genomehubs.org/view/idCriRanu1.1/dataset/CATOTT01/cumulative.

**Figure 5.**
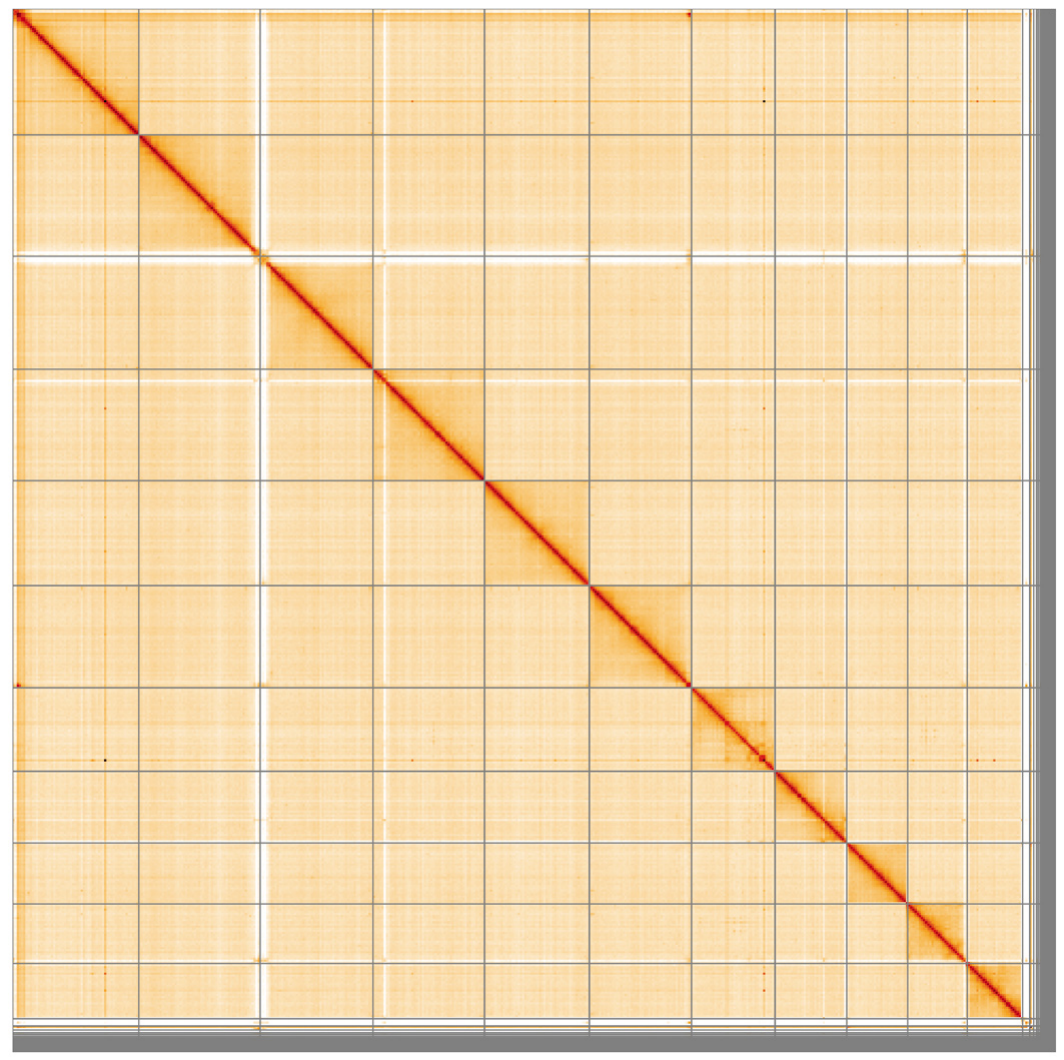
Genome assembly of *Ecdyonurus torrentis*, ieEcdTorr1.1: Hi-C contact map of the ieEcdTorr1.1 assembly, visualised using HiGlass. Chromosomes are shown in order of size from left to right and top to bottom. An interactive version of this figure may be viewed at https://genome-note-higlass.tol.sanger.ac.uk/l/?d=GE7fh8rORWifzZkFK1tYag.

The final assembly has a total length of 503.2 Mb in 121 sequence scaffolds with a scaffold N50 of 50.4 Mb ( Table 1). The snailplot in Figure 2 summarises the assembly statistics is shown in Figure 2, while the distribution of assembly scaffolds on GC proportion and coverage is shown in Figure 3. The cumulative assembly plot in Figure 4 shows curves for subsets of scaffolds assigned to different phyla. Most (97.25%) of the assembly sequence was assigned to 11 chromosomal-level scaffolds, representing 10 autosomes and the X sex chromosome. The X chromosome was identified by BUSCO synteny alignment to the curated male sample *Siphlonurus alternatus* (GCA_949825025.1). Chromosome-scale scaffolds confirmed by the Hi-C data are named in order of size ( Figure 5; Table 2). While not fully phased, the assembly deposited is of one haplotype. Contigs corresponding to the second haplotype have also been deposited. The mitochondrial genome was also assembled and can be found as a contig within the multifasta file of the genome submission.

**Table 1.** Genome data for *Ecdyonurus torrentis*, ieEcdTorr1.1.

Project accession data		
Assembly identifier	ieEcdTorr1.1	
Assembly release date	2023-04-08	
Species	*Ecdyonurus torrentis*	
Specimen	ieEcdTorr1	
NCBI taxonomy ID	2014018	
BioProject	PRJEB57424	
BioSample ID	SAMEA7520824	
Isolate information	ieEcdTorr1, female; posterior body (DNA sequencing and Hi-C scaffolding)	
Assembly metrics *		*Benchmark*
Consensus quality (QV)	51	*≥ 50*
*k*-mer completeness	99.97%	*≥ 95%*
BUSCO **	C:97.7%[S:96.2%,D:1.5%],F:1. 0%,M:1.2%,n:1,367	*C ≥ 95%*
Percentage of assembly mapped to chromosomes	97.25%	*≥ 95%*
Sex chromosomes	X chromosome	*localised homologous pairs*
Organelles	Mitochondrial genome assembled	*complete single alleles*
Raw data accessions		
PacificBiosciences SEQUEL II	ERR10480605, ERR10480606	
10X Genomics Illumina	ERR10489917, ERR10489918, ERR10489915, ERR10489916	
Hi-C Illumina	ERR10489919	
Genome assembly		
Assembly accession	GCA_949318235.1	
*Accession of alternate haplotype*	GCA_949318265.1	
Span (Mb)	503.2	
Number of contigs	1,012	
Contig N50 length (Mb)	1.0	
Number of scaffolds	121	
Scaffold N50 length (Mb)	50.4	
Longest scaffold (Mb)	60.6	

* Assembly metric benchmarks are adapted from column VGP-2020 of “Table 1: Proposed standards and metrics for defining genome assembly quality” from ( Rhie *et al.*, 2021). ** BUSCO scores based on the insecta_odb10 BUSCO set using v5.3.2. C = complete [S = single copy, D = duplicated], F = fragmented, M = missing, n = number of orthologues in comparison. A full set of BUSCO scores is available at https://blobtoolkit.genomehubs.org/view/idCriRanu1.1/dataset/CATOTT01/busco.

**Table 2.** Chromosomal pseudomolecules in the genome assembly of Ecdyonurus torrentis, ieEcdTorr1.

INSDC accession	Chromosome	Length (Mb)	GC%
OX439128.1	1	58.27	29.0
OX439129.1	2	54.23	28.5
OX439130.1	3	53.52	28.5
OX439131.1	4	50.41	28.5
OX439132.1	5	49.0	28.5
OX439133.1	6	40.19	28.5
OX439134.1	7	34.41	28.5
OX439136.1	9	29.25	27.5
OX439135.1	8	28.63	28.5
OX439137.1	10	26.49	28.5
OX439127.1	X	60.64	29.0
OX439138.1	MT	0.02	36.5

The estimated Quality Value (QV) of the final assembly is 51 with *k*-mer completeness of 99.97%, and the assembly has a BUSCO v5.3.2 completeness of 97.7% (single = 96.2%, duplicated = 1.5%), using the insecta_odb10 reference set ( *n* = 1,367).

Metadata for specimens, spectral estimates, sequencing runs, contaminants and pre-curation assembly statistics can be found at https://links.tol.sanger.ac.uk/species/2014018.

## Methods

### Sample acquisition and nucleic acid extraction

A female *Ecdyonurus torrentis* (specimen ID NHMUK014361706, ToLID ieEcdTorr1) was collected from River Rye, Yorkshire, UK (latitude 54.21, longitude –0.98) on 2019-05-06 using a kicknet. The specimen was collected and identified by Andrew Farr (independent researcher) and snap-frozen on dry ice.

DNA was extracted at the Tree of Life laboratory, Wellcome Sanger Institute (WSI). The ieEcdTorr1 sample was weighed and dissected on dry ice with tissue set aside for Hi-C sequencing. Posterior body tissue was cryogenically disrupted to a fine powder using a Covaris cryoPREP Automated Dry Pulveriser, receiving multiple impacts. High molecular weight (HMW) DNA was extracted using the Qiagen MagAttract HMW DNA extraction kit. Low molecular weight DNA was removed from a 20 ng aliquot of extracted DNA using the 0.8X AMpure XP purification kit prior to 10X Chromium sequencing; a minimum of 50 ng DNA was submitted for 10X sequencing. HMW DNA was sheared into an average fragment size of 12–20 kb in a Megaruptor 3 system with speed setting 30. Sheared DNA was purified by solid-phase reversible immobilisation using AMPure PB beads with a 1.8X ratio of beads to sample to remove the shorter fragments and concentrate the DNA sample. The concentration of the sheared and purified DNA was assessed using a Nanodrop spectrophotometer and Qubit Fluorometer and Qubit dsDNA High Sensitivity Assay kit. Fragment size distribution was evaluated by running the sample on the FemtoPulse system.

### Sequencing

Pacific Biosciences HiFi circular consensus and 10X Genomics read cloud DNA sequencing libraries were constructed according to the manufacturers’ instructions. DNA sequencing was performed by the Scientific Operations core at the WSI on Pacific Biosciences SEQUEL II (HiFi) and HiSeq X Ten (10X) instruments. Hi-C data were also generated from remaining tissue of ieEcdTorr1 using the Arima2 kit and sequenced on the Illumina NovaSeq 6000 instrument.

### Genome assembly, curation and evaluation

Assembly was carried out with Hifiasm ( Cheng *et al.*, 2021) and haplotypic duplication was identified and removed with purge_dups ( Guan *et al.*, 2020). The assembly was then scaffolded with Hi-C data ( Rao *et al.*, 2014) using YaHS ( Zhou *et al.*, 2023). The assembly was checked for contamination and corrected using the gEVAL system ( Chow *et al.*, 2016) as described previously ( Howe *et al.*, 2021). Manual curation was performed using gEVAL, HiGlass ( Kerpedjiev *et al.*, 2018) and Pretext ( Harry, 2022). The mitochondrial genome was assembled using MitoHiFi ( Uliano-Silva *et al.*, 2023), which runs MitoFinder ( Allio *et al.*, 2020) or MITOS ( Bernt *et al.*, 2013) and uses these annotations to select the final mitochondrial contig and to ensure the general quality of the sequence.

A Hi-C map for the final assembly was produced using bwa-mem2 ( Vasimuddin *et al.*, 2019) in the Cooler file format ( Abdennur & Mirny, 2020). To assess the assembly metrics, the *k*-mer completeness and QV consensus quality values were calculated in Merqury ( Rhie *et al.*, 2020). This work was done using Nextflow ( Di Tommaso *et al.*, 2017) DSL2 pipelines “sanger-tol/readmapping” ( Surana *et al.*, 2023a) and “sanger-tol/genomenote” ( Surana *et al.*, 2023b). The genome was analysed within the BlobToolKit environment ( Challis *et al.*, 2020) and BUSCO scores ( Manni *et al.*, 2021; Simão *et al.*, 2015) were calculated.

Table 3 contains a list of relevant software tool versions and sources.

**Table 3.** Software tools: versions and sources.

Software tool	Version	Source
BlobToolKit	4.1.7	https://github.com/blobtoolkit/blobtoolkit
BUSCO	5.3.2	https://gitlab.com/ezlab/busco
gEVAL	N/A	https://geval.org.uk/
Hifiasm	0.16.1-r375	https://github.com/chhylp123/hifiasm
HiGlass	1.11.6	https://github.com/higlass/higlass
Merqury	MerquryFK	https://github.com/thegenemyers/MERQURY.FK
MitoHiFi	2	https://github.com/marcelauliano/MitoHiFi
PretextView	0.2	https://github.com/wtsi-hpag/PretextView
purge_dups	1.2.3	https://github.com/dfguan/purge_dups
sanger-tol/genomenote	v1.0	https://github.com/sanger-tol/genomenote
sanger-tol/readmapping	1.1.0	https://github.com/sanger-tol/readmapping/tree/1.1.0
YaHS	1.1a.2	https://github.com/c-zhou/yahs

### Wellcome Sanger Institute – Legal and Governance

The materials that have contributed to this genome note have been supplied by a Darwin Tree of Life Partner. The submission of materials by a Darwin Tree of Life Partner is subject to the **‘Darwin Tree of Life Project Sampling Code of Practice’**, which can be found in full on the Darwin Tree of Life website here. By agreeing with and signing up to the Sampling Code of Practice, the Darwin Tree of Life Partner agrees they will meet the legal and ethical requirements and standards set out within this document in respect of all samples acquired for, and supplied to, the Darwin Tree of Life Project. 

Further, the Wellcome Sanger Institute employs a process whereby due diligence is carried out proportionate to the nature of the materials themselves, and the circumstances under which they have been/are to be collected and provided for use. The purpose of this is to address and mitigate any potential legal and/or ethical implications of receipt and use of the materials as part of the research project, and to ensure that in doing so we align with best practice wherever possible. The overarching areas of consideration are:

• Ethical review of provenance and sourcing of the material

• Legality of collection, transfer and use (national and international) 

Each transfer of samples is further undertaken according to a Research Collaboration Agreement or Material Transfer Agreement entered into by the Darwin Tree of Life Partner, Genome Research Limited (operating as the Wellcome Sanger Institute), and in some circumstances other Darwin Tree of Life collaborators.

## Data Availability

European Nucleotide Archive: *Ecdyonurus torrentis* (large brook dun). Accession number PRJEB57424; https://identifiers.org/ena.embl/PRJEB57424. ( Wellcome Sanger Institute, 2023) The genome sequence is released openly for reuse. The *Ecdyonurus torrentis* genome sequencing initiative is part of the Darwin Tree of Life (DToL) project. All raw sequence data and the assembly have been deposited in INSDC databases. The genome will be annotated using available RNA-Seq data and presented through the Ensembl pipeline at the European Bioinformatics Institute. Raw data and assembly accession identifiers are reported in Table 1.

## Author information

Members of the Natural History Museum Genome Acquisition Lab are listed here: https://doi.org/10.5281/zenodo.4790042.

Members of the Darwin Tree of Life Barcoding collective are listed here: https://doi.org/10.5281/zenodo.4893703.

Members of the Wellcome Sanger Institute Tree of Life programme are listed here: https://doi.org/10.5281/zenodo.4783585.

Members of Wellcome Sanger Institute Scientific Operations: DNA Pipelines collective are listed here: https://doi.org/10.5281/zenodo.4790455.

Members of the Tree of Life Core Informatics collective are listed here: https://doi.org/10.5281/zenodo.5013541.

Members of the Darwin Tree of Life Consortium are listed here: https://doi.org/10.5281/zenodo.4783558.

## References

[ref-1] Abdennur N Mirny LA : Cooler: Scalable storage for Hi-C data and other genomically labeled arrays. *Bioinformatics.* 2020;36(1):311–316. 10.1093/bioinformatics/btz540 31290943 PMC8205516

[ref-2] Allio R Schomaker‐Bastos A Romiguier J : MitoFinder: Efficient automated large‐scale extraction of mitogenomic data in target enrichment phylogenomics. *Mol Ecol Resour.* 2020;20(4):892–905. 10.1111/1755-0998.13160 32243090 PMC7497042

[ref-3] Bernt M Donath A Jühling F : MITOS: Improved *de novo* metazoan mitochondrial genome annotation. *Mol Phylogenet Evol.* 2013;69(2):313–319. 10.1016/j.ympev.2012.08.023 22982435

[ref-4] Buffagni A Cazzola M López-Rodríguez MJ : Distribution and Ecological Preferences of European Freshwater Organisms.Schmidt-Kloiber, A. and Hering, D. (eds.). Sofia-Moscow: Pensoft Publishers,2009;3. Reference Source

[ref-5] Challis R Richards E Rajan J : BlobToolKit - interactive quality assessment of genome assemblies. *G3 (Bethesda).* 2020;10(4):1361–1374. 10.1534/g3.119.400908 32071071 PMC7144090

[ref-6] Cheng H Concepcion GT Feng X : Haplotype-resolved *de novo* assembly using phased assembly graphs with hifiasm. *Nat Methods.* 2021;18(2):170–175. 10.1038/s41592-020-01056-5 33526886 PMC7961889

[ref-7] Chow A Brugger K Caccamo M : gEVAL — a web-based browser for evaluating genome assemblies. *Bioinformatics.* 2016;32(16):2508–2510. 10.1093/bioinformatics/btw159 27153597 PMC4978925

[ref-8] Di Tommaso P Chatzou M Floden EW : Nextflow enables reproducible computational workflows. *Nat Biotechnol.* 2017;35(4):316–319. 10.1038/nbt.3820 28398311

[ref-9] Elliott JM Humpesch UH Macan TT : Larvae of the British Ephemeroptera: A key with ecological notes.Scientific Publications of the Freshwater Biological Association,1988. Reference Source

[ref-10] GBIF Secretariat: *Ecdyonurus torrentis* Kimmins.1942, GBIF Backbone Taxonomy.2023; [Accessed 15 September 2023]. Reference Source

[ref-11] Guan D McCarthy SA Wood J : Identifying and removing haplotypic duplication in primary genome assemblies. *Bioinformatics.* 2020;36(9):2896–2898. 10.1093/bioinformatics/btaa025 31971576 PMC7203741

[ref-12] Harry E : PretextView (Paired REad TEXTure Viewer): A desktop application for viewing pretext contact maps. 2022; [Accessed 19 October 2022]. Reference Source

[ref-13] Howe K Chow W Collins J : Significantly improving the quality of genome assemblies through curation. *GigaScience.* Oxford University Press,2021;10(1): giaa153. 10.1093/gigascience/giaa153 33420778 PMC7794651

[ref-14] Kerpedjiev P Abdennur N Lekschas F : HiGlass: web-based visual exploration and analysis of genome interaction maps. *Genome Biol.* 2018;19(1): 125. 10.1186/s13059-018-1486-1 30143029 PMC6109259

[ref-15] Landa V : Development cycles of Central European Ephemeroptera and their interrelations. *Acta Entomol Bohemos.* 1968;65:276–284. Reference Source

[ref-16] Macan TT : The life histories and migrations of the Ephemeroptera in a stony stream. *Transactions of the Society for British Entomology.* 1957;12:129–156.

[ref-17] Manni M Berkeley MR Seppey M : BUSCO update: Novel and streamlined workflows along with broader and deeper phylogenetic coverage for scoring of eukaryotic, prokaryotic, and viral genomes. *Mol Biol Evol.* 2021;38(10):4647–4654. 10.1093/molbev/msab199 34320186 PMC8476166

[ref-18] Rao SSP Huntley MH Durand NC : A 3D map of the human genome at kilobase resolution reveals principles of chromatin looping. *Cell.* 2014;159(7):1665–1680. 10.1016/j.cell.2014.11.021 25497547 PMC5635824

[ref-19] Rhie A McCarthy SA Fedrigo O : Towards complete and error-free genome assemblies of all vertebrate species. *Nature.* 2021;592(7856):737–746. 10.1038/s41586-021-03451-0 33911273 PMC8081667

[ref-20] Rhie A Walenz BP Koren S : Merqury: Reference-free quality, completeness, and phasing assessment for genome assemblies. *Genome Biol.* 2020;21(1): 245. 10.1186/s13059-020-02134-9 32928274 PMC7488777

[ref-21] Simão FA Waterhouse RM Ioannidis P : BUSCO: assessing genome assembly and annotation completeness with single-copy orthologs. *Bioinformatics.* 2015;31(19):3210–3212. 10.1093/bioinformatics/btv351 26059717

[ref-22] Sowa R : Ecology and biogeography of mayflies (Ephemeroptera) of running waters in the Polish part of the Carpathians. 2. Life Cycles. *Acta Hydrobiologica.* 1975;17:319–353.

[ref-23] Sowa R : Le développement des Éphéméroptères de la rivière Dunajec six environs de Piening. In: *Proceedings of the 2nd International Conference on Ephemeroptera*.1979;125–131.

[ref-24] Surana P Muffato M Qi G : sanger-tol/readmapping: sanger-tol/readmapping v1.1.0 - Hebridean Black (1.1.0). *Zenodo.* 2023a; [Accessed 21 July 2023]. 10.5281/zenodo.7755665

[ref-25] Surana P Muffato M Sadasivan Baby C : sanger-tol/genomenote (v1.0.dev). *Zenodo.* 2023b; [Accessed 21 July 2023]. Reference Source

[ref-26] Uliano-Silva M Ferreira JGRN Krasheninnikova K : MitoHiFi: a python pipeline for mitochondrial genome assembly from PacBio high fidelity reads. *BMC Bioinformatics.* 2023;24(1): 288. 10.1186/s12859-023-05385-y 37464285 PMC10354987

[ref-27] Vasimuddin M Misra S Li H : Efficient Architecture-Aware Acceleration of BWA-MEM for Multicore Systems.In: *2019 IEEE International Parallel and Distributed Processing Symposium (IPDPS).*IEEE,2019;314–324. 10.1109/IPDPS.2019.00041

[ref-28] Wise EJ : Seasonal distribution and life histories of Ephemeroptera in a Northumbrian river. *Freshwater Biol.* 1980;10(2):101. 10.1111/j.1365-2427.1980.tb01185.x PMC7202231 32390674

[ref-30] Wellcome Sanger Institute: The genome sequence of the Large Brook Dun, *Ecdyonurus torrentis* (Kimmins, 1942). European Nucleotide Archive.[dataset], accession number PRJEB57424,2023. 10.12688/wellcomeopenres.20149.1 PMC10665603 37997584

[ref-29] Zhou C McCarthy SA Durbin R : YaHS: yet another Hi-C scaffolding tool. *Bioinformatics.* 2023;39(1): btac808. 10.1093/bioinformatics/btac808 36525368 PMC9848053

