# Supplementary material for: The genome sequence of the Large Brook Dun, Ecdyonurus torrentis (Kimmins, 1942)
Source: Wellcome Open Res. 2023 Oct 18;8:468. [Version 1] doi: 10.12688/wellcomeopenres.20149.1 (PMC10665603; doi:10.12688/wellcomeopenres.20149.1)
Supplement: Peer review history

# Reviewer report: Reviewer response for version 1

**DOI:** 10.21956/wellcomeopenres.22311.r69076

**Published:** 2023-11-22

**Authors:** Michael T. Monaghan

**Affiliations:** 1 Leibniz Institute of Freshwater Ecology and Inland Fisheries , Freie Universität Berlin, Berlin, Germany

This genome assembly of Ecdyonurus torrentis (Ephemeroptera, Heptageniidae) in an important contribution to our knowledge of Ephemeroptera genomes. The quality appears to be high based on the methodology used, the assembly statistics, and the busco score. It could also be noted that *E. torrentis* is likely a species complex, based on taxonomic work on the Ecdyonurinae, and that genomics data are likely to help our understanding of the evolution of this diverse group of insects.

Are sufficient details of methods and materials provided to allow replication by others?

Yes

Is the rationale for creating the dataset(s) clearly described?

Yes

Are the datasets clearly presented in a useable and accessible format?

Yes

Are the protocols appropriate and is the work technically sound?

Yes

Reviewer Expertise:

NA

I confirm that I have read this submission and believe that I have an appropriate level of expertise to confirm that it is of an acceptable scientific standard.

# Reviewer report: Reviewer response for version 1

**DOI:** 10.21956/wellcomeopenres.22311.r69073

**Published:** 2023-11-06

**Authors:** Isabel Almudi

**Affiliations:** 1 University of Barcelona, Barcelona, Spain

This data note presents the genome assembly of the mayfly *Ecdyonurus torrentis *from the Heptageniidae family of Ephemeroptera. Due to the relevance of this group for evolutionary and ecological studies, this genome assembly, and forthcoming Ephemeroptera genomes, will have great impact in these fields.

 In order to generate this assembly, an adult female was collected and processed. The genome is 503,2 megabases assembled in 10 autosomes, one X chromosome and a mitochondrial genome. The quality of the assembly is excellent with high completeness as shown by BUSCO. Overall, the methods employed are adequate and clearly listed in the note.

Are sufficient details of methods and materials provided to allow replication by others?

Yes

Is the rationale for creating the dataset(s) clearly described?

Yes

Are the datasets clearly presented in a useable and accessible format?

Yes

Are the protocols appropriate and is the work technically sound?

Yes

Reviewer Expertise:

insect genomics, evolution & development

I confirm that I have read this submission and believe that I have an appropriate level of expertise to confirm that it is of an acceptable scientific standard.
